# Lateral Acromioplasty has a Positive Impact on Rotator Cuff Repair in Patients with a Critical Shoulder Angle Greater than 35 Degrees

**DOI:** 10.3390/jcm9123950

**Published:** 2020-12-05

**Authors:** Edoardo Franceschetti, Edoardo Giovannetti de Sanctis, Alessio Palumbo, Riccardo Ranieri, Paola Casti, Arianna Mencattini, Nicola Maffulli, Francesco Franceschi

**Affiliations:** 1 Department of Orthopaedic and Trauma Surgery, Campus Biomedico University of Rome, Via Alvaro del Portillo 200, 00128 Rome, Italy; franceschetti.edo@gmail.com (E.F.); alessio.palumbo@hotmail.it (A.P.); 2 Department of Orthopaedics and Traumatology, Catholic University, Agostino Gemelli Hospital, 00168 Rome, Italy; 3 Department of Orthopaedic and Trauma Surgery, Humanitas Clinical and Research Center, Rozzano, 20089 Milan, Italy; riccardo.ranieri92@gmail.com; 4 Department of Electronics Engineering, University of Rome Tor Vergata, Via del Politecnico 1, 00133 Rome, Italy; casti@ing.uniroma2.it (P.C.); mencattini@ing.uniroma2.it (A.M.); 5 Department of Musculoskeletal Disorders, Via Salvador Allende, 43, 84081 Baronissi, Italy; n.maffulli@qmul.ac.uk; 6 Centre for Sports and Exercise Medicine, Barts and The London School of Medicine and Dentistry, Mile End Hospital, 275 Bancroft Road, London E1 4DG, UK; 7 School of Pharmacy and Bioengineering, Keele University School of Medicine, Thornburrow Drive, Stoke on Trent ST4, UK; 8 Department of Orthopaedic and Trauma Surgery, San Pietro Fatebenefratelli Hospital, Via Cassia 600, 00123 Rome, Italy; f.franceschi@unicampus.it

**Keywords:** rotator cuff tear, shoulder arthroscopy, shoulder, critical shoulder angle

## Abstract

Background: A Critical Shoulder Angle (CSA), evaluated on plain radiographs, greater than 35° is considered predictive of rotator cuff tears. The present prospective comparative study aimed, firstly, to develop a formula to calculate the amount of acromion that should be resected performing a lateral acromioplasty and, secondly, verify whether lateral acromioplasty to reduce the CSA associated with arthroscopic cuff repair decreased the rate of recurrence of the tears, and impacted favorably on clinical postoperative outcomes. Methods: Patients undergoing arthroscopic rotator cuff repair (RCR) for rotator cuff tears with a CSA greater than 35° were included in this study and divided into two groups, based on whether the CSA had been reduced by arthroscopic resection of the lateral portion of the acromion. A new mathematical formula was developed in order to quantify the amount of bone to be resected while performing the lateral acromioplasty. Patients with traumatic tears, previous surgery, osteoarthritis or plain radiographs, not classified as A1 according to Suter-Henninger, were excluded. Clinical and radiographic outcomes were assessed at a minimum of 2 years of follow-up considering the tear size. Results: 289 patients were included in this study. Thirty-seven were lost to follow-up. Group A (Lateral acromioplasty) patients included: 38 small tears, 30 medium tears, 28 large tears and 22 massive tears; Group B (control group) was composed of 40 small tears, 30 medium tears, 30 large tears and 23 massive tears. The Constants Score value and retear Rate were, respectively, significant higher (*p* = 0.007 and *p* = 0.004) and lower (*p* = 0.029 and *p* = 0.028) in Group A, both in the Small-and Medium-size subgroups. No complications were outlined. The mediolateral width of the acromion was reduced, according to the preoperatively calculated measure. Conclusion: Arthroscopic lateral acromioplasty decreased the CSA within the favorable range (30°–35°) in all patients treated, resecting the amount of bone predicted by the mathematical formula. Lateral acromioplasty is a safe and reproducible technique which may prevent recurrence of rotator cuff tears in patients with small and medium lesions. Level of evidence: II.

## 1. Introduction

Rotator cuff tears (RCTs) are common. Numerous factors, including age, activity level and smoking, have been associated with an increased risk of RCTs [1,2]. Furthermore, there is an association between scapular anatomy, regarding both acromion and glenoid morphology, and atraumatic rotator cuff tears (RCTs) [3,4].

Recently, the critical shoulder angle (CSA), a radiographic measure that accounts for both glenoid inclination and lateral extension of the acromion, has been proposed to identify patients at high risk of rotator cuff disease [5,6]. The CSA is produced between superior and inferior bone margins of the glenoid and the most lateral border of the acromion. Both larger (>35°) and smaller CSAs (<30°) are associated with an increased prevalence of, respectively, RCT and glenohumeral arthritis. 

Theoretically a CSA of >35° would require a lesser amount of deltoid force to produce superior migration of the humeral head. Therefore, the rotator cuff would work harder and exert a greater compensatory force to stabilize the humeral head within the glenoid during shoulder motion to establish an adequate fulcrum [7].

RCTs can be associated with a high CSA; when treated arthroscopically, such patients have a higher risk of retear and worse postoperative outcomes [8,9,10]. Hence, soft tissue repair alone does not seem to restore the high preoperative supraspinatus load seen with a higher CSA, predisponding to tendon retear [7].

Several authors [8,11,12,13,14] studied the effect of arthroscopic lateral acromioplasty on the CSA and its association with RCTs. The clinical outcomes of CSA correction on RCTs’ surgeries and retears are still debated [15]. Furthermore, the exact amount of acromion surface to be removed has not been described. The purpose of this study was to investigate whether arthroscopic lateral acromioplasty as an adjunct to rotator cuff repair (RCR) in patients with a CSA higher than 35° reliably decreases retear rate and whether it is associated with higher outcome scores. We also describe a mathematical formula, helping surgeons to obtain the desired postoperative CSA, based on three plain radiograph parameters.

Theoretically, a smaller CSA minimizes the biomechanical forces favoring superior translation of the humeral head, which may be advantageous after a RCR. Our hypothesis was that arthroscopic lateral acromioplasty would reliably decrease the retear rate without impacting on complication rate and would improve postoperative clinical outcomes.

## 2. Materials and Methods

This is a prospective comparative study approved by our local ethics committee. 

All patients were followed up longitudinally for a minimum of 24 months. All patients had a unilateral, degenerative full thickness RCT diagnosed by magnetic resonance (MR) and confirmed at surgery; a CSA higher than 35° based on preoperative plain radiographs; failure of at least 6 months of conservative treatment (including a shoulder rehabilitation physiotherapy program combined or not with injections or oral medications). In addition, both pre- and postoperative follow-up plain radiographs had to be classified as A1 according to the Suter/Henninger System (SH) in order to obtain high-quality AP (Antero-posterior) shoulder view, which is of primary importance for CSA measurement [16].

Patients with radiographic signs of osteoarthritis, traumatic or irreparable RCTs, isolated subscapularis tears, previous surgery, adhesive capsulitis, inflammatory disease, crystal arthropathy or with any deformity or bony irregularity of the glenoid or acromion obstructing the landmarks used to measure the CSA accurately, were excluded. A total of 73 patients out of 362 patients were excluded after applications of inclusion and exclusion criteria. A degenerative RCT was defined as a lesion with causes different from trauma. Considering that there are almost no definitive evidence-based data that facilitate discrimination between traumatic and nontraumatic [17], we consider, as degenerative RCT, lesions in patients who were not able to remember any trauma event with sudden worsening of symptoms and in patients with a history of shoulder symptoms who reported a worsening after a not unusual daily effort or minor trauma. In doubtful cases, MRI examinations were used to reach a definitive [17].

We chose to evaluate atraumatic tears to isolate the biomechanical effects of the CSA, avoiding confounding variables related to traumatic tears.

Fatty infiltration of the rotator cuff muscles greater than stage 3 according to the classification of Goutallier et al. [18] and chronic pseudoparalysis of active flexion were considered as irreparable tears. 

Two fellowship trained orthopedic surgeons with a special interest in shoulder arthroscopy performed all the surgical procedures. 

One surgeon agreed, for the purposes of the present study, not to perform the acromioplasty procedure, though both were trained and equally able to undertake it. All the patients in the present study were on the hospital waiting list, and were allocated to one or the other surgeon according to the day of admission. One surgeon performed all the acromioplasty procedures (Group A), and the other did not (Group B).

Demographic data such as age, gender and body mass index (BMI) were collected. The Constant Score [19] was the only one used and was collected at the initial visit (baseline) and at 24 months of follow-up. Abduction strength, according to the Constant and Murley Score, was measured as previously described with a validated electronic dynamometer and was assessed by a fully trained examiner different from the operating surgeons. 

Measurement of the preoperative and postoperative CSA was conducted on true anteroposterior radiographs according to the method described by Moor et al. [5]. The angle is formed by a line connecting the superior and inferior bony margins of the glenoid and a line drawn from the inferior bony margin of the glenoid to the most lateral border of the acromion. In addition, preoperative and postoperative lateral and axillary views were routinely obtained to exclude fracture of the acromion and heterotopic ossification.

Only patients with true anteroposterior plain radiographs classified as A1 according to the Suter/Henninger system [16] were included in this study. Alterations in the projection of the glenoid margin and the lateral extension of the acromion may consequently lead to errors in CSA measurement. Views beyond 5° anteversion, 8° retroversion, 15° flexion and 26° extension resulted in >2° deviation of the CSA compared to true AP [16]. Radiology technicians of our hospital were educated about carrying out it properly.

The value of 35° was chosen based on Moor et al.’s study [5], correlating it with an increased risk of RCT.

As deltoid tendon tear is one of the described complications for the lateral acromioplasty technique, we assessed the integrity of the deltoid. This included palpation of the origin, recording of any pain during contraction with the arm in neutral position and fibers continuity at MRIs, both at the initial visit and during the follow-up.

All patients underwent both preoperative MRI scans to diagnose and characterize the full thickness rotator cuff tear. A postoperative MRI scan was performed on all patients 12 months after the index procedure to evaluate the rotator cuff. Following examination of the preoperative MRI scan, the dimension of the tears was measured in mm in the T2-weighted parasagittal plane and classified according to Cofield et al. [20] as small <1 cm, medium 1–3 cm, large 3–5 cm, or massive >5 cm. Tendon healing on postoperative MRI scans was analyzed with the Sugaya classification [21], and types I–III were catalogued as healed (no retear) while types IV and V were catalogued as retorn tendons (retear) [21].

All imaging studies were analyzed by one author, who was blinded to the names of the patients.

To avoid removing more acromion than necessary and decrease CSA more than 30°, we defined, analyzing true AP plain radiographs, a mathematical relationship between CSA and other parameters. Given its simplicity, the geometrical expression is reliable for a large group of patients and can be used preoperatively to reduce the CSA. 

**Figure 1:**
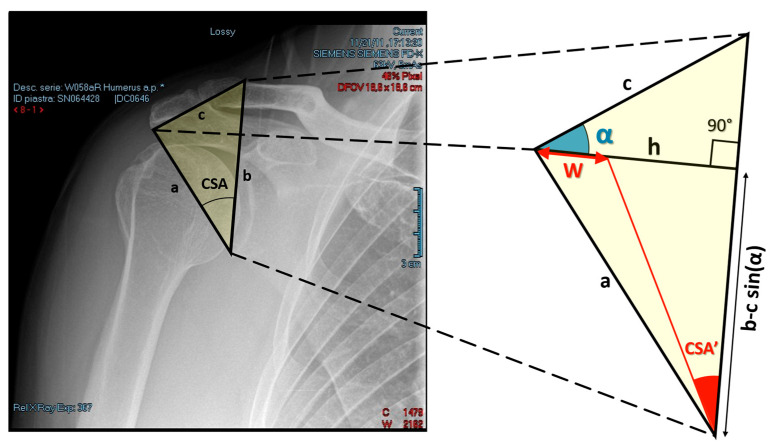
Geometrical derivation of the formula to estimate the amount of lateral resection, w, to obtain a desired postoperative CSA, namely CSA’. Parameters a, b, and c are the radiographic measures needed for the computation. CSA: critical shoulder angle.

The triangle, as shown in Figure 1, on which the formula relies on is produced by the intersection of three lines. Each edge of the triangle represents one of the three radiographic measures of interest.

- Edge a: the segment drawn from the inferior margin of the glenoid to the lateral aspect of the acromion;
- Edge b: the segment passing through the superior margin of the glenoid fossa, connecting the inferior margin of the glenoid fossa to the superior margin of the clavicle;
- Edge c: the segment connecting the lateral aspect of the acromion to the superior margin of the clavicle defined through edge b.

Edges a and b define the preoperative CSA, as shown in Figure 1.

Using the Heron’s formula, the triangle’s area, A, can be expressed in terms of its three sides as

(1) $$A=\sqrt{P\left(P-a\right)\left(P-b\right)\left(P-c\right)},$$

where *P* is the half perimeter of the triangle, namely, P=(a+b+c)/2.

The relation

(2) h=2A/b

defines the height of the triangle with respect to edge b. Next, consider α, the angle formed by the intersection of lines c and h (see Figure 1). α can be calculated with the following formula:

(3) $$\alpha =\cos^{-1}\left(h/c\right)$$

The tangent of an angle can be derived dividing the length of the opposite side by the length of the adjacent side. With reference to Figure 1, the tangent of the postoperative CSA, or CSA’, can be calculated as

(4) $$\tan\left(CSA^{\prime }\right)=\frac{h-w}{b-c\sin\alpha }$$

where w is the amount of lateral resection of the acromial bone during lateral acromioplasty. The final objective is to analytically determine the amount of lateral resection, w, to obtain the desired CSA’. This is achieved by using the previous relationship to calculate w with the following formula

(5) $$w=h-\tan\left(CSA^{\prime }\right)\left[b-c\sin\left(\alpha \right)\right]$$

From the mathematical formula above, it is clear that the amount of lateral resection only depends on the desired postoperative CSA, i.e., CSA’, if the three radiographic measures, i.e., margins a, b, and c, are derived directly from the radiograms. Note that the values of h and α are computed using, respectively, Equations (2) and (3), with A defined by Equation (1). 

The amount of lateral resection, $W^{\prime }$, that will determine a postoperative CSA within the interval (30°–35°) is given, using Equation (1), as

(6) $$W^{\prime }=w\left(CSA^{\prime }=32.5{}^{\circ}\right)\pm \varepsilon$$

with $\varepsilon =\left[w\left(CSA^{\prime }=30{}^{\circ}\right)-w\left(CSA^{\prime }=35{}^{\circ}\right)\right]/2$.

For the case in Figure 1, the radiographic measures, derived directly from the radiogram, are 69.97 mm, 77.36 mm, and 45.48 mm, corresponding to parameters a, b, and c, respectively. The estimated amount of lateral resection, i.e., $w\left(CSA^{\prime }=32.5{}^{\circ}\right),$ is 4.36 ± 3.50 mm. The obtained value was calculated using Equation (1) with $CSA^{\prime }=32.5{}^{\circ}$ ($CSA^{\prime }=\;$0.57 rad), h = 40.65 mm and sin(α) = 0.45, as derived from Equations (1)–(3). The only needed radiographic measures were a, b, and c.

The estimated dispersion of the amount of lateral resection is referred to the CSA range (30°–35°). The indication on the amount of lateral resection provided to the surgeon in order to reduce the CSA at the level of 32.5° was 4.4 mm.

To test the reliability of the proposed formula, a Monte Carlo analysis was performed, which takes into account the effects of erroneous radiographic measurements of parameters a, b, and c on the computed amount of lateral resection. M = 10^6^ random sampling from three Gaussian probability distributions with mean a, b, and c, respectively, and standard deviation equal to 1 mm, which correspond to an error of about 5% on each parameter, were performed. The obtained confidence interval of 1.76–6.93 mm for a 95% coverage probability is within the interval [w(CSA′ = 30°) − w(CSA′ = 35°)] corresponding to the desired range of CSA, i.e., 30°–35°.

The mathematical formula was implemented in Microsoft Excel. This could be done because a straightforward relationship between the three radiographic measures, named a, b, and c, and the amount of lateral resection was geometrically derived. By means of an Rx viewer commonly used in clinical practice, surgeons can determine the three radiographic measures with point-to-point distance measurements. Subsequently, by inserting the three obtained numerical values, i.e., a, b, and c, in the Excel table, the indication of the amount of the lateral resection is directly provided.

### 2.1. Surgical Procedure

All patients were operated on in the lateral position under general or interscalene anesthesia by two trained fellowship orthopaedic surgeons.

Through a posterior arthroscopic viewing portal, an intra-articular examination confirmed the full-thickness RCT. Then, a resector is introduced through an anterolateral portal to clean the footprint. When all soft tissues are removed from the lateral margin, in Group A’s patients, the surgeon translates the value calculated preoperatively to a distance to the undersurface of the acromion with a calibrated probe, and marks it with an electrocautery. Then, the part of the acromion laterally to that mark is progressively resected, starting on the bursal side of it and working in an inferosuperior, anteroposterior and mediolateral direction, so that the white tendon tissue of the undersurface of the deltoid origin begins to be visible from the subacromial space. The mediolateral width of the acromion is thereby reduced, according to the preoperative calculation. Whatever the theoretically calculated amount of bone, the upper limit for a safe resection was considered as half of acromion in mediolateral width; however, this limit was never reached while resecting the amount of bone predicted by the formula. In Group B, lateral acromioplasty was not performed; bursectomy and occasional removal of gross spurs were performed only for arthroscopic viewing reasons. The tendon is then repaired to the footprint with a single-row technique. Biceps tenodesis or tenotomy was performed in case of biceps instability or biceps lesions. The incisions are closed in a routine fashion, and the shoulder is positioned in an abduction brace for 4 weeks.

### 2.2. Statistical Analysis

Unpaired *t*-tests and Chi-squared tests were respectively used to compare parametric and nonparametric variables. Mean and standard deviation (SD) were calculated. When 3 or more groups were compared, Kruskall–Wallis tests were used. Strength of association between two variables were measured using the Spearman’s Rho test for rank correlations of ordinal variables and the Pearson test for linear associations between continuous variables.

A P value of <0.05 was regarded as statistically significant. Microsoft Excel version 15.0 (Microsoft, Redmond, WA, USA) was used to analyze the data.

## 3. Results

**Figure 2:**
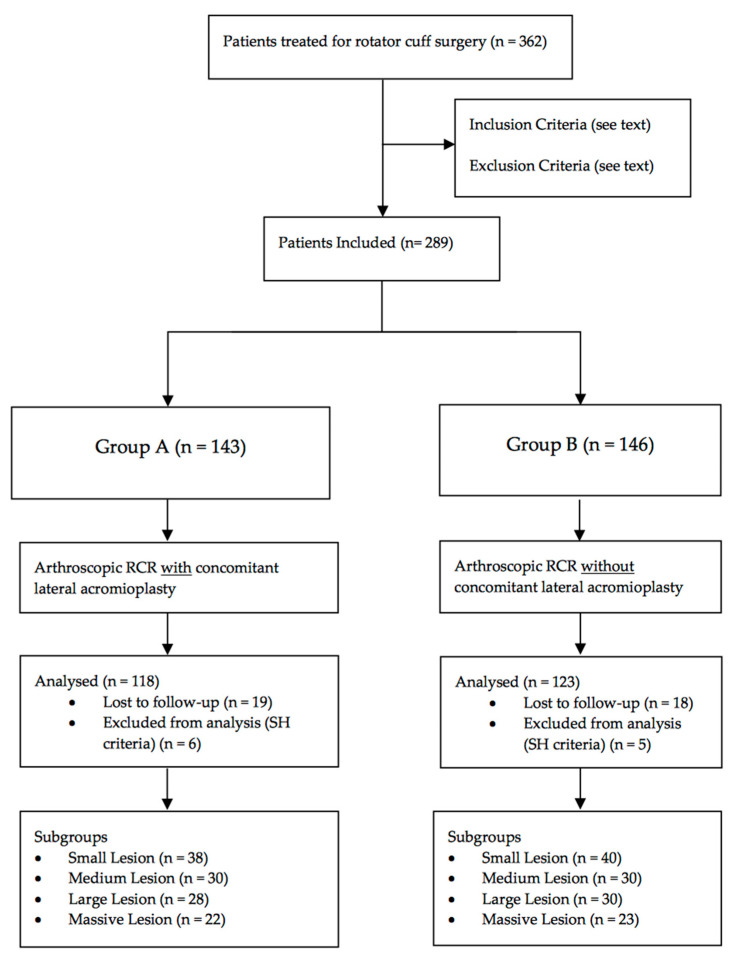
Flow Diagram of the current study.

From January 2015 to December 2017, 143 patients underwent arthroscopic RCR with concomitant lateral acromioplasty (Group A), and 146 patients underwent arthroscopic RCR without concomitant lateral acromioplasty (Group B) (Figure 2). All had a unilateral, degenerative full-thickness RCT and a CSA higher than 35°. Of these patients, 37 (13%) were lost to follow-up. Then, eleven patients were excluded from analysis as their postoperative plain radiographs were not classified as A1.

The remaining were divided into 4 subgroups according to the tear size. In the first class, 38 had a small lesion, 30 a medium lesion, 28 a large lesion and 22 a massive lesion. In the second category, 40 had a small tear, 30 a medium tear, 30 a large tear, and 23 a massive tear. The minimum follow-up was 24 months, the mean follow-up was 27.81 months.

The demographic characteristics are summarized in Table 1, and radiographic data and clinical outcomes are summarized in Table 2. Biceps tenodesis or tenotomy was performed in 37 (31.4%) and 45 shoulders (36.6%), respectively, in groups A and B, which was not statistically different (*p =* 0.392).

Results were analyzed, comparing the subgroups, based on the tear size. 

The result of the Kruskall–Wallis test (*p* < 0.00001) indicates that preoperative CSA values relative to the four subgroups did not come from the same distribution. In particular, multiple comparison (post hoc) test with the Tukey–Kramer method indicated significant differences in preoperative CSA between small and large tears (*p* < 0.001), small and massive tears (*p* < 0.00000001), medium and massive tears (*p* < 0.0001), large and massive tears (*p* < 0.05). For the remaining comparisons, *p* = 0.25 and *p* = 0.23 were obtained between small and medium tears, and between medium and large tears, respectively. 

The mediolateral width of the acromion was reduced, according to the preoperatively calculated measure, by an average of about 6 mm. There was a significant reduction in CSA for each tear size (5.15 ± 1.71, *p* < 0.00001; 5.97 ± 2.15, *p* < 0.00001; 6.21 ± 1.81, *p* < 0.00001; 7.59 ± 2.17, *p* < 0.00001; respectively, for small, medium, large and massive tears, Group A). Within each tear size category, groups A and B did not differ significantly for demographic and for preoperative Constant Score and CSA value (Table 2). A significant improvement in Constant Score after arthroscopic cuff repair was found in all patients regardless of the tear size and acromioplasty. Lateral acromioplasty, performed only in Group A, significantly decreased the critical shoulder angle in each subgroup. However, differences could be highlighted. 

Small tear lesions in Group A have a significantly higher Constant Score value and lower retear rate than in Group B (*p* = *0*.007 and *p* = 0.029). Furthermore, medium tear lesions in Group A have a significantly higher Constant Score value and lower retear rate than in Group B (*p* = 0.004 and *p* = 0.028). There was no significant correlation between amount of CSA correction and gain in Constant Score (Pearson, R (36) = −0.1561, *p* = 0.0916).

No significant differences related to Constant Score and retear rate were found in large and massive tear subgroups. During follow-up, clinically and at final MRI, no macroscopic injury to the deltoid muscle insertion nor acromion fracture were identified. 

## 4. Discussion

The most important finding of this study was that lateral acromioplasty in addition to rotator cuff repair in patients with small and medium tears and with a CSA greater than 35° is associated with better postoperative Constant Score and decreased risk of retear.

Our purpose, on the efficacy of lateral acromioplasty in diminishing rotator cuff repair failures and modifying postoperative clinical outcomes, was confirmed partially.

Although plenty of studies have focused on whether the reduction of CSA diminishes the risk of developing an RCT in patients with subacromial impingement and tendon retear in patients who underwent rotator cuff repair, this topic is still debated.

In the study by Balke et al. [22], shoulders with degenerative tears showed a narrower subacromial space and a more lateral extension of the acromion compared to traumatic tears.

Garcia et al. [10] were the first to demonstrate association between CSA and the risk of retear and poor outcomes after arthroscopic rotator cuff repair. Furthermore, in their study, values > 38° both seemed to be a more consistent predictor of rotator cuff disease and increased the risk of retear after repair, contrary to the literature average CSA values correlated with rotator cuff disease, which vary from 35° to 39°. 

Zumstein et al^.^ [23] and Schreider et al. [9] identified a wide lateral extension of the acromion as a risk factor for retearing after prior arthroscopic repair. This may suggest that soft tissue repair alone, without restoring elevated preoperative supraspinatus load seen with higher CSA, does not seem to mitigate the risk of tendon retear. However, often higher retear rates have not been associated with poor outcome scores, suggesting caution in interpreting those imaging results [24,25,26].

As an insufficient CSA reduction was associated with a higher rate of retear and lower abduction strength with healed tendon, an accurate acromioplasty gains importance and has to be performed in a controlled fashion according to accurate preoperative planning.

In contrast, few clinical studies [27,28] found that the CSA were not a predictor of patient-reported outcomes or recurrence RCT after primary arthroscopic repair. 

Altintas et al. [11], Katthagen et al. [14] and Gerber et al. [8] confirmed in vivo the preliminary in cadaver studies, which suggested that arthroscopic lateral acromioplasty can be performed without significant risk to the deltoid origin.

Recently, Degen [15] criticized past studies on this topic, for both the lack of association between CSA and clinical outcome measures after cuff repair, and cutoffs considered as significant for this variable, asking for more evidence to support correction of CSA with lateral acromioplasty and objective data correlating CSA with outcomes. Indeed, even small differences in patient position, ranging from 5° to 8° of ante/retroversion, can result in more than 2° changes in the CSA measurement. Therefore, differences in CSA between groups could have resulted from inaccurate patient positioning. 

Gerber et al. [8] reported improved abduction strength with reduction of CSA after rotator cuff repair, a higher risk of retear for larger (preoperative and) postoperative CSAs, and that lateral acromioplasty can be performed without significant damage to the deltoid tendon. However, they failed to identify improvements in objective outcome measures; the exact amount of required lateral resection was not measured precisely at the beginning of their study; no instrument/surgical technique was used to measure and resect the exact amount of lateral acromion needed.

To tackle the limits of previous studies, the measurement of the CSA was performed only on true AP Suter/Henninger A1 plain radiographs. Then, to avoid removing too small or too large an amount of acromial bone, we defined a formula estimating the amount of lateral acromion resection that the surgeon needs to perform obtaining a postoperative CSA within the range 30–35°, as suggested by previous studies, and confirmed by the present one. 

Our hypothesis was confirmed in part, as only patients with small and medium CSA have a biomechanical advantage after RCR, resulting in significant improved functional scores and lower retear rates. Despite the Constant Score being significantly higher in small and medium lesions of Group A, the difference was approximately 3 points, which is below the MCID (minimal clinical importance difference) [29]. It may be possible that a clinical difference would present with a longer follow-up period.

The reduction of a CSA was effectively and safely achievable through an arthroscopic lateral acromioplasty; indeed, after the procedure, the CSA decreased significantly in the whole study group, with no complications recorded. 

This study has limitations. We have not been able to minimize the loss to follow-up, which was higher than 5%. We did not evaluate and record how cuff and deltoid tendons modify on MRI during follow-up, such as scar forming healing, fatty infiltration, small and partial dehiscence. The Cofield (MRI) and Suter/Henninger (plain radiographs) imaging classifications, as well as the CSA calculation, were not performed by a musculoskeletal radiologist, but a single orthopedic surgeon.

Patients’ plain radiographs, wrongly classified as A1, could have been included in this study, altering the results. We did not record which, among the rotator cuff tendons, were torn and/or tore again and the retear etiology. Another limit of this study is the use of only one shoulder score. Our surgical technique of translating the preoperatively calculated value of the amount of bone to be resected to the inferior surface of the acromion could be not very precise. Further studies should evaluate and compare the value estimated by our formula with the amount of bone resected, focusing on precision of the procedure. 

We are aware that the evidence reported in the present article is not as strong as what would have been produced in a level I randomised controlled trial. Given our clinical set-up, this quasi-randomised study design was readily feasible, and the results obtained are compelling.

This study also has several strengths. This is the first attempt to characterize an association between the CSA and rotator cuff tears, divided by width. Furthermore, higher clinical outcomes after RCR, when carrying out a lateral acromioplasty, even if limited to small and medium tears, have been outlined. The inclusion of only A1 plain radiographs and the formula produced allow to measure precisely preoperatively the exact amount of required lateral resection. 

## 5. Conclusions 

Arthroscopic lateral acromioplasty performed in addition to arthroscopic RCR, with single-row repair, can reduce the CSA without significantly compromising the deltoid origin, decreasing the risk of retear and improving clinical outcomes in patients with small and medium tears. Those findings were not confirmed in patients with large and massive tears. This technique might be able to change the course of this condition by preventing external impingement and optimizing the deltoid force vector. 

Our formula is aimed at a normal CSA which is epidemiologically associated with neither osteoarthritis nor rotator cuff disease, but we are aware that the ideal CSA has not been established to be between 30° and 35°.

The results of this study should be viewed as exploratory. Further high-quality investigations, required to confirm our results and definitely answer the role of lateral acromioplasty on outcomes, should ideally use more than one score, elucidate the best and precise CSA value between 30 and 35 degrees, develop a new surgical technique resecting the acromion more precisely, according to preoperative calculation, and evaluate our new formula.

## Figures and Tables

**Table 1:** Demographics and perioperative data of the 2 groups and 4 subgroups of patients.

Tear Size		Gender (F/M)	BMI (Mean ± SD)	Age (Mean ± SD)	Follow Up Duration mo. (Mean ± SD)
**Small**					
	Group A	22/16	26.95 ± 5.39	57 ± 9.65	28.23 ± 4.49
	Group B	24/16	27.15 ± 6.16	56.25 ± 9.18	28.425 ± 4.59
	*p*	0.850	0.817	0.726	0.855
**Medium**					
	Group A	17/13	26.93 ± 5.60	57.23 ± 7.92	28.45 ± 4.39
	Group B	16/14	27.33 ± 6.19	58 ± 8.41	27.93 ± 4.40
	*p*	0.796	0.793	0.717	0.655
**Large**					
	Group A	17/11	27.64 ± 6.34	61.75 ± 5.16	28.25 ± 4.65
	Group B	17/13	27.07 ± 6.31	58.83 ± 6.39	27.86 ± 4.50
	*p*	0.750	0.730	0.060	0.751
**Massive**					
	Group A	10/12	26.68 ± 5.92	63 ± 5.36	26.68 ± 3.98
	Group B	12/11	28.35 ± 6.17	62.09 ± 5.12	27.13 ± 4.18
	*p*	0.652	0.360	0.562	0.714

BMI: Body Mass Index; mo: months; SD: Standard Deviation.

**Table 2:** Comparison of the influence of Lateral Acromioplasty on Constant–Murley Score preoperatively and postoperatively and retear rate in 4 subgroups of patients, characterized by the tear size.

Tear Size		CSA (Mean ± SD)		Constant (Mean ± SD)		Retear Rate (R/T)
		Pre-op	Post-op	Pre-op	Post-op	
**Small**						
	Group A	38.44° ± 2.14	33.29° ± 1.21	53.55 ± 5.32	86.37 ± 4.05	2/38
	Group B	38.05° ± 1.84	36.85° ± 1.87	54.5 ± 6.22	83.52 ± 5.02	9/40
	*p*	0.383		0.471	0.007	0.029
**Medium**						
	Group A	39.1° ± 2.00	33.13° ± 0.97	50.16 ± 4.73	79.17 ± 4.68	3/30
	Group B	38.67° ± 1.94	37.3° ± 2.05	50.63 ± 3.01	76.03 ± 3.44	10/30
	*p*	0.398		0.650	0.004	0.028
**Large**						
	Group A	39.43° ± 1.91	33.21° ± 1.19	45.78 ± 4.86	73.18 ± 5.41	8/28
	Group B	39.8° ± 1.83	38.83° ± 2.07	46.43 ± 4.24	71.83 ± 4.71	12/30
	*p*	0.453		0.650	0.318	0.360
**Massive**						
	Group A	40.5° ± 1.79	32.91° ± 1.06	39.86 ± 4.33	64.5 ± 6.07	10/22
	Group B	41° ± 1.57	39.65° ± 1.82	41 ± 4.60	63.43 ± 5.59	12/23
	*p*	0.856		0.953	0.938	0.0652

SD: Standard Deviation; CSA: Critical Shoulder Angle; R/T: Retear/Total of patients.
